# Supplementary material for: Comparative genomics provides new insights into the diversity, physiology, and sexuality of the only industrially exploited tremellomycete: Phaffia rhodozyma
Source: BMC Genomics. 2016 Nov 9;17:901. doi: 10.1186/s12864-016-3244-7 (PMC5103461; doi:10.1186/s12864-016-3244-7)
Supplement: Additional file 6: — List of orphan genes with links to PFAM (related to Additional file 1: Table S1). (ZIP 1428 kb) [file 12864_2016_3244_MOESM6_ESM.zip › PFAM_UNIQUE/G01507_P.html]

#                                                                            --- full sequence --- -------------- this domain -------------   hmm coord   ali coord   env coord
  
# target name        accession   tlen query name           accession   qlen   E-value  score  bias   #  of  c-Evalue  i-Evalue  score  bias  from    to  from    to  from    to  acc description of target
  
#------------------- ---------- ----- -------------------- ---------- ----- --------- ------ ----- --- --- --------- --------- ------ ----- ----- ----- ----- ----- ----- ----- ---- ---------------------
  
G01507\_P             -            337 ATP-synt\_E           PF05680.7     90       1.4    7.6   5.1   1   2     0.021        21    3.8   6.5    25    85    97   155    94   170 0.75 - ATP synthase E chain  
G01507\_P             -            337 ATP-synt\_E           PF05680.7     90       1.4    7.6   5.1   2   2       2.1   2.1e+03   -2.6   0.0    14    26   268   280   254   283 0.78 - ATP synthase E chain  
G01507\_P             -            337 DLIC                 PF05783.6    472     0.023   12.1   2.7   1   1   1.6e-05     0.032   11.6   2.7   355   463    46   152    31   160 0.83 - Dynein light intermediate chain (DLIC)  
G01507\_P             -            337 MAP7                 PF05672.6    171      0.13   10.4   1.6   1   1   0.00083      0.21    9.6   1.6    22    81    99   159    82   195 0.85 - MAP7 (E-MAP-115) family
